# Knowledge, Attitudes, and Practices Associated With Diabetic Foot Prevention Among Rural Adults With Diabetes in North China

**DOI:** 10.3389/fpubh.2022.876105

**Published:** 2022-05-20

**Authors:** Huimin Jia, Xiaocheng Wang, Jingmin Cheng

**Affiliations:** ^1^School of Public Health, Shanxi Medical University, Taiyuan, China; ^2^School of Management, Shanxi Medical University, Taiyuan, China

**Keywords:** knowledge, attitudes, practices, rural patients, diabetic foot

## Abstract

**Background:**

The diabetic foot is a global threat to public health because it can result in infection and amputation, as well as cause the patient to experience considerable pain and incur financial costs. The condition of patients with diabetic foot in North China is distinguished by more severe local ulcers, a worse prognosis, and a longer duration of disease than that of patients with diabetic foot in the south. Through appropriate preventive measures, the diabetic foot can be effectively avoided. This study assesses the existing knowledge, attitudes and practices associated with diabetic foot prevention among adults with diabetes living in rural areas of North China.

**Method:**

This cross-sectional survey included 1,080 rural adults from North China, cluster sampled 12 villages and surveyed diabetic patients without diabetic foot who participated in community diabetes management. The self-administered knowledge and attitude questionnaire and the Chinese version of the Nottingham Assessment of Functional Foot-care Questionnaire were used.

**Result:**

Of the 1,080 subjects, 51.6% received moderate knowledge scores, 63.9% had a positive attitude and 71.4% received poor practice scores. In terms of knowledge, parameters of knowledge about foot examinations and treatment of foot problems showed the lowest scores. In terms of practice, in line with the results of the low knowledge score, parameters of the pursuit of medical treatment for foot problems and routine foot examinations were associated with the lowest scores. Multiple regression analysis revealed that participants who were current smokers (β: −0.049, 95% CI: −0.088 to −0.011) had lower knowledge scores than those who never smoke; participants who were current smokers (β: −0.818, 95% CI: −1.067 to −0.569) and past smokers (β: −0.299, 95% CI: −0.485 to −0.112) had lower attitude scores than those who had never smoked; participants who had higher knowledge scores (β: 1.964, 95% CI: 1.572–2.356) achieved higher scores on attitudes; women had better practice scores than men (β: 0.180, 95% CI: 0.122–0.239); patients with a long diabetes duration (6–10 years) had better practice scores than those who had a short diabetes duration (<2 years; β: 0.072, 95% CI: 0.012–0.131). Knowledge (β: 0.130, 95% CI: 0.001–0.258) and attitudes (β: 0.268, 95% CI: 0.249–0.287) were significantly associated with good practices.

**Conclusions:**

Increasing knowledge regarding diabetic foot would help instill positive attitudes and cultivate better practices toward diabetic foot prevention. The results of this study may help guide future promotional resources to those groups most in need, which may help lower the incidence of diabetic foot among adults in North China.

## Introduction

Diabetes is a metabolic disorder characterized by high blood glucose levels and is one of the most common chronic non-communicable diseases worldwide (1). According to the International Diabetes Federation Diabetes Atlas 10^th^ edition, an estimated 537 million people worldwide had diabetes in 2021, with that figure expected to rise to 643 million by 2030 (2). There are many patients with diabetes in China, and its prevalence rate has rapidly increased recently (3).

The prognosis of diabetes is perturbing due to long-term hyperglycemia leading to chronic damage and dysfunction of various tissues, especially the eyes, kidneys, heart, blood vessels and nerves (4). Diabetic foot is one of the most difficult complications to treat among all the complications of diabetes (5). Diabetic foot ulcers (DFU) are associated with high morbidity and mortality globally (6). A diabetic limb is amputated every 20 s according to estimates (7). In a Chinese tertiary hospital, the overall amputation rate among patients with diabetic foot ulcers was reported to be 21.5% (8), and mortality associated with lower extremity arterial diseases in patients with diabetes exceeded that associated with most cancers (except lung cancer, pancreatic cancer and others) (9). Furthermore, diabetic foot frequently necessitates extended hospitalization, which raises costs (10). As a result, the diabetic foot is regarded as one of the leading causes of disability and death in diabetes patients and a major public health issue imposing a significant socio-economic burden (7). Therefore, policymakers and academic researchers are focusing more on diabetic foot prevention.

Diabetic foot is preventable (11); early identification of and implementation of timely intervention against the risk factors of diabetic foot is critical for its prevention and treatment. In developed countries, growing evidence regarding diabetic foot treatment has revealed that relatively simple and low-cost interventions can reduce amputation rates by up to 85% (12). Peripheral neuropathy, peripheral vascular disease, abnormal plantar pressure, poor blood glucose control, and smoking are all risk factors for diabetic foot ulcers, and they all play a role in the disease's pathophysiology (13). Furthermore, studies have shown that age, gender, education level, lifestyle and socio-economic status are also important factors (14).

Previous research has also investigated the psychological factors of diabetic foot prevention. Palaya et al. (15) noted that self-efficacy influences diabetic patients' self-management behavior. Guo et al. (16) discovered that due to long-term disease treatment and management, patients may develop diabetes-related psychological distress such as depression and anxiety, including medical consultation-related distress, change-in-condition-related distress, and emotional burden-related distress, which can prevent patients from dealing with foot ulcer management and glycaemic control. Laopoulou et al. point out that the social support that patients receive from different sources can help them develop better self-management perseverance and help facilitate self-management practices (17). Over the last two decades, a large number of diabetic foot KAP studies have been conducted focused on identifying barriers to foot-care and improving foot care (18–20). Most studies agree that having more knowledge and positive attitudes toward diabetic foot care can help promote good diabetic foot-care practices (18, 21). KAP-related studies can be used to improve patient foot care and to develop appropriate diabetic foot interventions.

Although there is an increasing number of studies on the diabetic foot, little is known about knowledge, attitudes and practices related to diabetic foot prevention among adults with diabetes living in rural areas of North China. Compared with those in southern China, patients with diabetic foot patients in North China were reported have a longer course of podiatry and a worse prognosis. Patients in the south are more affected by vascular and inflammatory factors, whereas those in the north are more affected by factors not only affected by hematology and vascular lesions but also restricted by economic conditions (22). Optimal foot-care practices may be the most cost-effective method for prevention or detecting diabetic foot complications, particularly in resource constrained areas (23). Furthermore, although studies have examined the impact of demographic variables on KAP of diabetic foot prevention, differences may exist between populations due to geographical, economic, and cultural factors. As a result, we need to understand the current status of KAP in diabetic foot prevention in patients with diabetes in rural North China, as well as the factors that influence them.

Understanding the current situation, identifying gaps, and improving policy require assessing the KAP for diabetic foot prevention among rural northerners. Therefore, the purpose of this study was to examine the knowledge on diabetic foot prevention, attitudes toward diabetic foot prevention, and foot-care practices among rural adults with diabetes in North China. We also aimed to identify the association between knowledge, attitudes and practices, and sociodemographic and clinical variables.

## Materials and Methods

This cross-sectional study included rural adults with diabetes living in North China. Ethics approval was obtained from the Scientific Ethics Review Board of Shanxi Medical University (Code: 2021010). Participation in this study was voluntary, and informed consent was obtained from all participants.

### Participants

We used the following Cochrane formula to determine the minimum sample size of this study:


(1) $$N\;=\;\frac{Z^{2}pq}{d^{2}}$$

where *N* = sample size, *p* = prevalence of diabetes in Chinese adults; *q* = 1 – *p*; *Z* = standard normal deviation, usually set at 1.96, corresponding to the 95% confidence interval; and *d* = degree of accuracy desired, set at 0.02 in this study. Therefore, the minimum sample size calculated was 1,043. Assuming that 3% of the questionnaire answers would be incomplete, the sample size was finally determined to be 1,080.

All samples were selected using a multistage, stratified cluster sampling design (provinces, cities, or villages were selected as strata), and the clusters were selected from each strata. From October 2021 to January 2022, four cities were randomly selected in North China based on different geographical characteristics: Huaibei of Anhui Province, Baoding of Hebei Province, Liaoyang of Liaoning Province and Yantai of Shandong Province. Three villages were selected from each of the four cities. Participants were recruited based on the management records of each village. The inclusion criteria were patients with diabetes aged ≥18 years, living in rural areas of North China, clinically diagnosed with type 1 and type 2 diabetes for >6 months and with no foot ulcers. All diabetic patients in the selected villages who met the inclusion criteria and agreed to participate in the survey were included in the study. Patients who could not answer questions because of their mental state or showed severe clinical symptoms were excluded.

### Questionnaires

The questionnaire comprises four sections. The first section is regarding the sociodemographic characteristics, including gender, age, marital status and education status, and clinical characteristics, including diabetes duration and smoking status, of the patients.

The second section comprises 11 questions regarding knowledge on diabetic foot prevention knowledge which was developed based on the researchers' knowledge and experience and published information (24). This section was designed to investigate the two dimensions of foot-care knowledge, i.e. risk factors of diabetic foot (five items) and foot examinations and treatment of foot problems (six items), using a two-point scale (0 = false and not sure, 1 = correct answer).

The third section of the questionnaire includes nine items to measure people's attitudes toward diabetic foot prevention. The questionnaire was designed to investigate three dimensions of foot-care attitudes, i.e. susceptibility to diabetic foot (two items), the importance of diabetic foot prevention (five items), and the initiatives related to diabetic foot prevention (two items), using a five-point Likert scale (1 = strongly disagree, 2 = disagree, 3 = neutral, 4 = agree, 5 = strongly agree).

Finally, the fourth section of the questionnaire measures people's foot-related self-care practices. The Chinese version of the Nottingham Assessment of Functional Foot-care Questionnaire developed by Jing Li's team has been proven to be a valid and reliable method of assessing diabetic foot-care practices; therefore, it was used in this study (25). This questionnaire included 24 items, including five dimensions, such as daily foot examination (three items), foot cleaning (four items), foot protection (five items), choosing shoes and socks (nine items), and the behavior of seeking medical treatment for foot problems (three items). Among these, eight items follow reverse scoring. Additionally, the participants were asked to rate the frequency of performing the abovementioned practices on a four-point Likert scale, with higher scores indicating better foot self-care practices.

### Statistical Analyses

Descriptive data are presented as means, standard deviations, and absolute frequencies and percentages depending on whether the variables were continuous or categorical. The sum score of each outcome was assessed based on Bloom's cut-off point (26). Knowledge was classified into the low level (<60%; 0–6 scores), moderate level (60–80%; 7–8 scores) and high level (>80%; 9–11 scores). Attitudes were classified into negative attitudes (<60%; 0–26 scores), neutral attitudes (60–80%; 27–36 scores) and positive attitudes (>80%; 37–45 scores). Practices were classified into the poor (<60%; 0–57 scores), moderate (60–80%; 58–76 scores), and good (>80%; 77–96 scores) levels. Pearson's correlation analysis was also used to determine the correlations between knowledge, attitudes and practices of diabetic foot prevention. Association of sociodemographic and clinical characteristics with knowledge, attitude, and practice was assessed using *t*-tests and analysis of variance (ANOVA). More specifically, *t*-test was used to assess the significant differences between two dependent variables. One-way ANOVA test was used to assess the significance of differences among three and more dependent variables. Multiple linear regression models were conducted to analyse predictor variables that were associated with the knowledge, attitude, and foot self-care practice scores. Variables that proved to be statistically significant in univariate analysis were further subjected to multiple linear regressions as well as the progressive incorporation of knowledge, and attitudes according to KAP theory. Specifically, gender and smoking status were entered as predictors in the regression model, with knowledge scores as the dependent variable. Gender, age, education status, diabetes duration, smoking status, and knowledge scores were entered as predictors in the regression model, with attitude scores as the dependent variable. Gender, duration of disease, smoking status, knowledge scores, and attitude scores were entered as predictors in the regression model, with practice scores as the dependent variable. All statistical analyses were performed using the Statistical Package for Social Sciences (SPSS; v.25).

## Results

### Sociodemographic and Clinical Characteristics and Sources of Information on Diabetic Foot

A total of 1,080 participants completed the questionnaire; of these, 556 respondents (51.5%) were men, 595 (55.1%) were aged >60 years, and 866 (80.2%) were married. Regarding the education status, 648 (60%) of the 1,080 participants received primary school education or lower education and 176 (16.3) received a high school degree or higher education. More than one-third (*n* = 435, 40.3%) of the respondents had diabetes that lasted >10 years and 109 (10.1%) were current smokers. The sociodemographic and clinical characteristics of the study population are shown in Table 1.

**Table 1:** Sociodemographic and clinical characteristics of the study population (*n* = 1,080).

**Variables**	**Categories**	**Frequency**	**Percent (%)**
Gender	Men	556	51.5
	Women	524	48.5
Age (years)	≤ 40	55	5.1
	41–60	430	39.8
	≥61	595	55.1
Marital status	Married	866	80.2
	Divorced	25	2.3
	Widowed	189	17.5
Education status	Primary school education or below	648	60.0
	Junior high school degree	256	23.7
	High school degree or above	176	16.3
Diabetes duration	<2 years	126	11.7
	2–5 years	220	20.4
	6–10 years	299	27.7
	>10 years	435	40.3
Smoking status	Never	541	50.1
	Past smoker	430	39.8
	Current smoker	109	10.1

### Knowledge, Attitudes and Practices of Diabetic Foot Prevention

#### Knowledge on Diabetic Foot Prevention

The highest possible score for all knowledge-related questions was 11. Based on the data of the 1,080 participants, the knowledge score ranged between 2 and 11 (mean ± standard deviation, 7.4 ± 1.5), indicating an overall knowledge level of 67.3%. Further, 63.5% participants scored low on knowledge on foot examinations and treatment of foot problems (Table 2). The deficiencies are mainly reflected by the considerations that smoking is unimportant for preventing diabetic foot, that people with diabetes do not take long to heal from foot injuries and foot problems, and that scratching feet when the skin is dry; and that blisters and calluses can be treated by patients themselves.

**Table 2:** Knowledge scores of diabetic foot prevention.

**Dimensions**	**Total** **score**	***M*** **(SD)**	**Score** **rate (%)**
Risk factors of diabetic foot	5	3.59 (1.00)	71.8
Foot examination and treatment for foot problems	6	3.81 (1.08)	63.5
Total score	11	7.40 (1.53)	67.3

#### Attitudes Toward Diabetic Foot Prevention

Regarding attitudes, the highest possible score for all attitude-related questions was 45. Based on the data of the 1,080 participants, the attitude score ranged between 11 and 44 (mean ± standard deviation, 33.93 ± 9.0). It was observed that most participants (690, 63.9%) had a positive attitude. Among the three dimensions of attitude-related questions, the score for susceptibility to diabetic foot was the highest, that for the importance of diabetic foot prevention was lower, and that for the initiative of diabetic foot prevention was the lowest (Table 3).

**Table 3:** Attitude scores of diabetic foot prevention.

**Dimensions**	**Total score**	***M*** **(SD)**	**Score rate (%)**
Susceptibility to diabetic foot	10	7.61 (2.07)	76.1
Importance of diabetic foot prevention	25	18.79 (5.14)	75.2
Initiative of diabetic foot prevention	10	7.43 (2.17)	74.3
Total score	45	33.83 (8.99)	75.2

#### Practices of Foot Self-Care

Regarding practices of foot self-care, 771 (71.4%) participants scored poorly. Among the five dimensions of foot-care practice, the scores for the behavior of seeking medical treatment for foot problems was the lowest (40.4%) and that for daily foot examination was also low (42.3%), which was lower than the score rates for the other three dimensions (Table 4).

**Table 4:** Practice scores of diabetic foot prevention.

**Dimensions**	**Total score**	***M*** **(SD)**	**Score rate (%)**
Daily foot examination	12	5.07 (1.56)	42.3
Foot cleaning	16	10.69 (3.67)	66.8
Foot protection	20	13.88 (4.03)	69.4
Choosing shoes and socks	36	19.42 (5.74)	54.0
Seeking medical treatment for foot problems	12	4.85 (1.21)	40.4
Total score	96	53.91 (10.11)	56.3

### Univariate Analysis of Diabetic Foot Prevention Knowledge, Attitudes, and Foot-Care Practices

Univariate analysis revealed that the mean scores for knowledge were significantly different among participants based on their gender (*t*-test, *P* < 0.05) and smoking status (ANOVA, *P* < 0.05). The mean attitude score was significantly different among participants based on their gender (*t*-test, *P* < 0.05), age, education status, diabetes duration and smoking status (ANOVA, *P* < 0.05; Table 5). The mean practice scores were significantly different among participants based on their gender (*t*-test, *P* < 0.05), diabetes duration, and smoking status (ANOVA, *P* < 0.05; Table 5).

**Table 5:** Univariate analysis of diabetic foot prevention knowledge, attitudes, and foot self-care practices.

**Variables**	**Mean of** **knowledge**	**SD**	***P-*** **Value**	**Mean of** **attitude**	**SD**	***P-*** **Value**	**Mean of** **practice**	**SD**	***P-*** **Value**
**Gender**									
(1) Male	0.66	0.14	0.048^a^	3.56	1.10	<0.001^a^	2.10	0.36	<0.001^a^
(2) Female	0.69	0.13		3.97	0.81		2.40	0.42	
**Age**									
(1) 40 and below	0.65	0.15	0.352^b^	3.41	1.20	<0.001^b^	2.13	0.42	0.068^b^
(2) 41–60	0.67	0.15		3.67	1.06		2.24	0.47	
(3) ≥61	0.68	0.13		3.86^1^, ^2^	0.91		2.26	0.38	
**Marital status**									
(1) Married	0.67	0.14	0.214^b^	3.75	1.00	0.676^b^	2.25	0.42	0.757^b^
(2) Divorced	0.63	0.12		3.68	1.08		2.28	0.57	
(3) Widowed	0.68	0.14		3.81	0.94		2.23	0.40	
**Education status**									
(1) Primary school education and below	0.67	0.14	0.866^b^	3.83	0.93	0.006^b^	2.23	0.37	0.345^b^
(2) Junior high school degree	0.67	0.14		3.68	1.05^1^		2.27	0.51	
(3) High school and above	0.67	0.15		3.60	1.11^1^, ^2^		2.27	0.47	
**Diabetes duration**									
(1) <2 years	0.66	0.15	0.782^b^	3.61	1.10	0.002^b^	2.19	0.46	<0.001^b^
(2) 2–5 years	0.67	0.14		3.58	1.12		2.15	0.37	
(3) 6–10 years	0.67	0.14		3.87^1^, ^2^	0.87		2.33^1^, ^2^	0.44	
(4) >10 years	0.68	0.14		3.82^1^, ^2^	0.95		2.56^2^, ^3^	0.41	
**Smoking status**									
(1) Never	0.69	0.13	<0.001^b^	4.00	0.77	<0.001^b^	2.40	0.41	<0.001^b^
(2) Past smoker	0.67	0.14		3.64^1^	1.06		2.13^1^	0.37	
(3) Current smoker	0.63^1^, ^2^	0.16		3.03^1^, ^2^	1.23		1.98^1^, ^2^	0.39	

a *t-test*.

b *ANOVA test*.

*Post-hoc multiple comparisons were performed using the LSD-t method for each variable with significant ANOVA results,*

1 *statistically significant difference compared to the first group;*

2 *statistically significant difference compared to the second group;*

3 *statistically significant difference compared to the third group*.

### Correlation Between Knowledge, Attitudes, and Practices of Diabetic Foot Prevention

A correlation test indicated a direct and significant correlation between knowledge and attitudes (*P* < 0.01, *r* = 0.309), knowledge and practices (*P* < 0.01, *r* = 0.257), and attitudes and practices (*P* < 0.01, *r* = 0.700).

### Multiple Factors Analysis of Diabetic Foot Prevention Knowledge, Attitudes and Foot Self-Care Practices

As presented in Table 6, the multivariable linear regression suggested that smoking status remained statistically significant in the final multivariable linear regression analysis (*F* = 5.355, *P* = 0.001). The current smokers had lower knowledge scores than those who never smoked (β: −0.049, 95% CI: −0.088 to −0.011, *P* = 0.011).

**Table 6:** Multiple linear regression results of knowledge, attitudes, and practices toward diabetic foot prevention.

**Dependent**	**Independent**	**Unstandardized**		**Standardized**	***t***	***P-*** **Value**	**95% CI**	
**variable**	**variable**	**coefficients**		**coefficients**				
		**β**	**SE**				**Lower**	**Upper**
Knowledge	Constant	0.670	0.028		24.114	<0.001	0.615	0.724
	**Gender**							
	Male	Ref.						
	Female	0.008	0.014	0.029	0.570	0.569	−0.020	0.036
	**Smoking status**							
	Never	Ref.						
	Past smoker	−0.010	0.014	−0.035	−0.686	0.493	−0.038	0.018
	Current smoker	−0.049	0.019	−0.107	−2.552	0.011	−0.087	−0.011
Attitude	Constant	2.426	0.271		8.939	<0.001	1.984	2.959
	**Gender**							
	Male	Ref.						
	Female	0.033	0.095	0.017	0.347	0.728	−0.153	0.219
	**Age**							
	40 and below	Ref.						
	41–60	0.076	0.136	0.038	0.562	0.574	−0.190	0.343
	≥61	0.233	0.165	0.117	1.412	0.158	−0.091	0.557
	**Education status**							
	Primary school and below	Ref.						
	Junior high school degree	0.012	0.095	0.005	0.128	0.898	−0.175	0.199
	High school and above	−0.018	0.118	−0.007	−0.149	0.882	−0.249	0.214
	**Diabetes duration**							
	<2 years	Ref.						
	2–5 years	−0.086	0.102	−0.035	−0.847	0.397	−0.285	0.113
	6–10 years	0.080	0.113	0.036	0.707	0.480	−0.141	0.301
	>10 years	0.002	0.123	0.001	0.012	0.990	−0.239	0.242
	**Smoking status**							
	Never	Ref.						
	Past smoker	−0.299	0.095	−0.148	−3.147	0.002	−0.485	−0.112
	Current smoker	−0.818	0.127	−0.249	−6.450	<0.001	−1.067	−0.569
	**Knowledge**	1.964	0.200	0.275	9.829	<0.001	1.572	2.356
Practice	Constant	0.876	0.078		11.198	<0.001	0.722	1.029
	**Gender**							
	Male	Ref.						
	Female	0.180	0.030	0.214	6.090	<0.001	0.122	0.239
	**Diabetes duration**							
	<2 years	Ref.						
	2–5 years	−0.044	0.032	−0.042	−1.390	0.165	−0.106	0.018
	6–10 years	0.072	0.030	0.076	2.369	0.018	0.012	0.131
	>10 years	0.015	0.029	0.017	0.515	0.607	−0.042	0.071
	**Smoking status**							
	Never	Ref.						
	Past smoker	−0.027	0.030	−0.032	−0.914	0.361	−0.086	0.031
	Current smoker	0.015	0.040	0.011	0.374	0.708	−0.064	0.095
	**Knowledge**	0.130	0.065	0.043	1.979	0.048	0.001	0.258
	**Attitude**	0.268	0.010	0.632	27.996	<0.001	0.249	0.287

*The adjusted R square of knowledge model was 0.012. The adjusted R square of attitude model was 0.171. The adjusted R square of practice model was 0.547*.

Smoking status and knowledge showed statistically significant predictive capability for attitude scores. These two independent variables could explain 17.1% variation of attitude (*F*= 21.234, *P* < 0.001). Specifically, the attitude scores of current smokers (β: −0.818, 95% CI: −1.067 to −0.569, *P* < 0.001) and past smokers (β: −0.299, 95% CI: −0.485 to −0.112, *P* = 0.002) were lower than those of never smokers. And knowledge positively affected attitude toward diabetic foot prevention (β: 1.964, 95% CI: 1.572–2.356, *P* < 0.001), the attitude score increases with the increase of knowledge score (Table 6).

The results indicated that gender, diabetes duration, knowledge and attitude played a part in practice of foot care; these four independent variables could explain 54.7% variation of practice (*F* = 164.038, *P* < 0.001). Specifically, women had better practice scores than men (β: 0.180, 95% CI: 0.122–0.239, *P* < 0.001). Patients with a long diabetes duration (6–10 years) had better practice scores than those with a short diabetes duration (<2 years; β: 0.072, 95% CI: 0.012–0.131, *P* = 0.018). Knowledge (β: 0.130, 95% CI: 0.001–0.258, *P* = 0.048) and attitudes (β: 0.268, 95% CI: 0.249–0.287, *P* < 0.001) positively affect practices (Table 6).

## Discussion

Late complications of diabetes, especially diabetic foot, may lead to amputation, resulting in functional decline, increased economic burden on patients and a sharp decline in the patients' quality of life. Therefore, preventing diabetic foot is necessary. To the best of our knowledge, this is the first survey of its kind to be conducted in North China to determine the knowledge, attitudes, and foot-care practices regarding diabetic foot prevention among rural adults with diabetes. As measured by our survey, 23.3% of patients with diabetes have good knowledge on diabetic foot prevention and most have a positive attitude toward preventing diabetic foot. However, only 3% of patients with diabetes followed good diabetic foot prevention practices. The practice scores were lower than knowledge scores as revealed by the questionnaire, thus reflecting poor compliance with good self-care practices. Our results were comparable to those of other studies where the practice scores were always lower than knowledge scores (27, 28).

### Present Knowledge on Diabetic Foot Prevention

In our study, 56.1% of the participants had moderate and 25.1% had poor knowledge on diabetic foot. These results are in accordance with those of a previous study conducted in 144 hospitals across 31 Chinese provinces. Li et al. (29) reported that most patients with type 2 diabetes have a medium level of knowledge. Another study in Iran also reported similar results (30). Smoking is a significant risk factor for peripheral artery disease, which is directly related to the development of the diabetic foot; therefore, quitting smoking is crucial for preventing diabetic foot (31). Scratching of feet when the skin is dry may increase the risk of skin ulceration (32). Due to the influence of long-term hyperglycemia, oxidative stress, and various vascular and neurological complications, wound healing in patients with diabetes is usually delayed, resulting in chronic ulcers and diabetic foot. Notably, callus removal should be performed by professionally trained diabetic podiatrists as untimely treatment or improper management can lead to local or general infection, gangrene in serious cases, and even to amputation (33).

### Present Attitudes Toward Diabetic Foot Prevention

The results of the present study demonstrated that the majority of the participants (63.9%) had a positive attitude toward prevention of diabetic foot. Analysis of the three dimensions of attitude indicated that most patients are well aware of their susceptibility of diabetic foot but pay little attention to its prevention, and lack motivation to take preventive measures. A possible reason for this may be that diabetic foot complications develop slowly, which leads patients to not realize the serious consequences in a short period of time, leading to them having a laidback attitude, patients think that they will not have diabetic foot or that complications will not occur in the near future, which subsequently leads to patients not taking measures to prevent diabetic foot.

### Present Foot Self-Care Practices

The results of our study indicated that the foot self-care practices among the population of the rural areas in North China are concerning. Many patients treated corns, calluses and wounds on their own. This is consistent with the findings of studies conducted around the world (34–36). Patients chose to self-treat their foot problems, possibly due to a lack of foot-care knowledge or poor availability of medical facilities in rural areas.

The results of this study also indicated that daily foot examination behavior was poor. This is also in line with the results of other domestic (37) and international studies (34). Sun et al. (37) observed that some patients believe that asymptomatic feet do not require daily examination and, as a result, do not value the daily examination of feet and shoes before wearing them. Poor implementation of the daily examination dimension may be related to the patients' lack of knowledge on foot care for diabetic foot prevention. Furthermore, foot self-care necessitates long-term commitment, and checking feet and shoes every day can be repetitive and boring with no discernible effect in the short term.

### Factors Influencing Foot-Care Knowledge, Attitudes and Practices

Our study examined the factors influencing the level of foot prevention knowledge, attitudes and practices among the participants. We found that current smokers had lower levels of knowledge and poor attitudes toward diabetic foot prevention than those who never smoked. According to Khamseh et al. (30), possible explanations for this include lower health literacy among smokers and reluctance to accept new information, making it more difficult for them to understand the complex disease mechanisms of the diabetic foot and the means of prevention offered; this makes them less motivated to take prevention measures (38). This could be a warning sign as smokers are more likely to develop foot complications such as ulcers and amputation in the future.

Our results revealed that rural women scored higher than men in terms of foot self-care practices related to diabetic foot prevention. Similar findings were also reported in studies conducted by Rossaneis et al. in Brazil. The difference observed may be attributed to the fact that men have lower levels of health literacy and concern for their health than women. Women pay more attention to the signs and symptoms of diseases, are more concerned about their body image, and have difficulty accepting the inability to walk properly and physical defects caused by diabetic foot, whereas men are often reluctant to admit their health problems and seek professional care (39). Another study on men's self-perceived health confirmed that most men did not seek medical care even after being diagnosed with a chronic disease owing to a lack of time during the working days, their schedules not coinciding with the working hours of health services, lack of severe symptoms, or because they faced more challenges in accessing medical services than women (40). As a result, women practiced better foot self-care than men. According to a meta-analysis, men with diabetic foot have roughly one and a half the amputation risk than women with diabetic foot (41). To tackle this, diabetic men should receive adequate health education.

The duration of diabetes influenced the mean practice scores; patients with a longer diabetes duration performed better in foot self-care practice. This is consistent with the findings of previous research (29, 42, 43). It is possible that patients having diabetes for a long duration were more likely to have repetitive educational sessions, which may favor their attitude and practice scores. However, patients having diabetes for a short duration may have less of an opportunity to receive foot self-care education. Therefore, individualized and systematic education should be developed and guidance should be provided according to disease duration to improve the self-care practice related to diabetic foot prevention in patients with diabetes.

Our study revealed that educational status had no impact on the foot self-care practice scores. Several studies found a significant association between education status and diabetic foot-care practice levels (18, 44). This difference can be explained by the lack of adequate promotion of diabetic foot awareness in our study population. Both highly and poorly educated patients were inadequately informed about diabetic foot prevention.

Our results also indicated that knowledge positively affected attitudes and knowledge and attitudes positively affected practices. The better the patients' knowledge on foot care, the more positive was their attitudes toward preventing diabetic foot. Additionally, the more active measures that patients took to prevent diabetic feet, the better they cared for their feet. These results are consistent with previous studies (42, 45). According to the KAP theory, the relationship between knowledge, attitudes and practices is progressive. Knowledge and information are the foundation for developing positive and correct beliefs and attitudes for changing health-related behavior.

Notably, the findings indicate that participants' diabetes knowledge did not translate into action to prevent foot problems. This implies that the intervention should shift from traditional education to critical education, i.e. the focus of education shifts from a purely knowledge-based domain to a concrete behaviors-based level. The mindsponge mechanism, which illustrates how a person can absorb new values and eject waning values conditionally based on contexts (46), suggests that specific individual characteristics and the interaction between the individual and the environment need to be considered when designing educational interventions (47). Specifically, when exposed to new information, people judge whether to keep it or discard it based on perceived value, the closer the information is to mindset, the more likely it is to be accepted. Accepted information becomes part of one's belief system and can influence subsequent decisions.

Health education aims to provide information to promote behavioral change to enhance health and quality of life. It is necessary to develop facilities for the patients to reinforce and maintain the desired behavior and to make the patients with diabetes willing participants in the educational process. This necessitates a collaborative effort between the hospital, health workers, health education teams, patients, and their families (48, 49). When caring for diabetic patients, healthcare professionals should first assess their patients' knowledge and skills in diabetic foot care, particularly their ability to deal with foot problems daily. Moreover, health education teams target patients to provide them with the knowledge they need while assessing their willingness to use effective learning methods. Subsequently, a special foot clinic is recommended for hospitals and correct treatment methods are recommended for rural family doctors to increase the likelihood of patients with diabetes seeking medical help when they develop foot problems. These measures make it easier for patients with diabetes to seek medical help. Further, for patients to develop the habit of daily foot examinations, they are advised to organically integrate various care practices with their daily habits, which require the help and support of family members. As family members are a closely related group of patients with similar daily lifestyles, involving them in management helps reduce isolation and boredom as well as improve patients' self-care practices. To sum up, it is suggested that patients' diabetic foot-care practices be improved in the future through well-targeted knowledge education, increased motivation to prevent diabetic foot, facilitated medical visits, and an environment that encourages family foot care.

### Limitations

Foot self-care practices were determined through a self-reported questionnaire, which may have response and recall biases. Prospective studies with larger sample sizes are warranted to explore the soci-cultural, clinical, and psychological factors that influence foot-care behavior, and further qualitative studies may be needed to explore additional influencing factors.

## Conclusions

Patients with diabetes in rural areas of North China have poor diabetic foot prevention knowledge and foot-care behavior. We discovered that knowledge, attitude, gender, and duration of diabetes significantly influenced patients' practice of foot care. Considering the severity of the problem of diabetic foot in North China and the low level of knowledge and foot self-care practice, it is necessary to strengthen the education of diabetic foot prevention knowledge among adult patients in rural areas of North China. The findings of this study may help guide future promotional resources to those groups most in need, which may help reduce the occurrence of diabetic foot among adults in North China. Structured programs need to be planned to improve the knowledge, attitudes, and practices of diabetic foot prevention. Education should be differentiated by gender, diabetes duration, and smoking status.

## Data Availability Statement

The raw data supporting the conclusions of this article will be made available by the authors, without undue reservation.

## Ethics Statement

The studies involving human participants were reviewed and approved by Scientific Ethics Review Board of Shanxi Medical University. Written informed consent for participation was not required for this study in accordance with the national legislation and the institutional requirements.

## Author Contributions

JC, HJ, and XW contributed to conception and design of the study. JC funded the study. HJ and XW participated in field study, organized the database, and performed the statistical analysis. HJ wrote the first draft of the manuscript. XW wrote sections of the manuscript. All authors contributed to manuscript revision, read, and approved the submitted version.

## Conflict of Interest

The authors declare that the research was conducted in the absence of any commercial or financial relationships that could be construed as a potential conflict of interest.

## Publisher's Note

All claims expressed in this article are solely those of the authors and do not necessarily represent those of their affiliated organizations, or those of the publisher, the editors and the reviewers. Any product that may be evaluated in this article, or claim that may be made by its manufacturer, is not guaranteed or endorsed by the publisher.
